# The role of visual crowding in eye movements during reading: Effects of text spacing

**DOI:** 10.3758/s13414-023-02787-1

**Published:** 2023-10-11

**Authors:** Tzu-Yao Chiu, Denis Drieghe

**Affiliations:** 1 School of Psychology, University of Southampton, Southampton, UK; 2 Department of Psychology, The Ohio State University, 225 Psychology Building, 1835 Neil Avenue, Columbus, OH 43210 USA

**Keywords:** Reading, Eye movements, Visual crowding, Parafoveal processing

## Abstract

Visual crowding, generally defined as the deleterious influence of clutter on visual discrimination, is a form of inhibitory interaction between nearby objects. While the role of crowding in reading has been established in psychophysics research using rapid serial visual presentation (RSVP) paradigms, how crowding affects additional processes involved in natural reading, including parafoveal processing and saccade targeting, remains unclear. The current study investigated crowding effects on reading via two eye-tracking experiments. Experiment 1 was a sentence-reading experiment incorporating an eye-contingent boundary change in which reader’s parafoveal processing was quantified through comparing reading times after valid or invalid information was presented in the parafovea. Letter spacing was jointly manipulated to compare how crowding affects parafoveal processing. Experiment 2 was a passage-reading experiment with a line spacing manipulation. In addition to replicating previously observed letter spacing effects on global reading parameters (i.e., more but shorter fixations with wider spacing), Experiment 1 found an interaction between preview validity and letter spacing indicating that the efficiency of parafoveal processing was constrained by crowding and visual acuity. Experiment 2 found reliable but subtle influences of line spacing. Participants had shorter fixation durations, higher skipping probabilities, and less accurate return sweeps when line spacing was increased. In addition to extending the literature on the role of crowding to reading in ecologically valid scenarios, the current results inform future research on characterizing the influence of crowding in natural reading and comparing effects of crowding across reader populations.

## Introduction

**Fig. 1:**
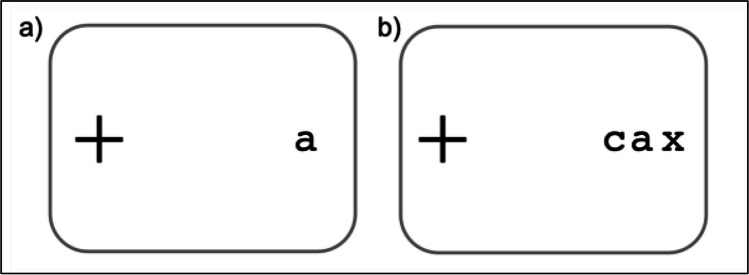
Demonstration of visual crowding

The human visual system is foveated, such that information presented at the center (i.e., the fovea) is received with high resolution whereas objects presented in the periphery (i.e., peripheral vision) are perceived as more jumbled and indistinct. This feature, originally thought to be caused by the rapid drop of visual acuity outside the fovea (Anstis, 1974), was later revealed to be largely attributable to peripheral vision’s susceptibility to visual crowding (Latham & Whitaker, 1996; Rosenholtz, 2016; Strasburger, 2020). Visual crowding, generally defined as the deleterious influence of clutter on visual discrimination, is a form of inhibitory interaction between nearby objects (Levi, 2008; Pelli & Tillman, 2008). As an example, it should be easy to recognize the letter “a” whilst looking at the fixation cross in Fig. 1a. However, when flankers are present, as in Fig. 1b, it becomes more difficult to identify the letter “a” despite it being at the same distance to the fixation cross. In psychophysical studies, crowding was commonly quantified using *critical spacing*: The target-flanker distance at which the observer’s task performance (e.g., letter recognition accuracy) reaches a certain criterion (e.g., 0.75 letter recognition accuracy). Critically, there has been an abundance of evidence showing that critical spacing is across studies approximately 0.5 times target eccentricity, a phenomenon known as *Bouma’s law* (Bouma, 1970, 1973; but see Whitney & Levi, 2011, for a discussion).

As the visual environments humans live in are inherently cluttered, crowding is ubiquitous in spatial vision and serves as a fundamental sensory limitation on visual cognition. Reading in particular, may be prone to constraints of crowding since written text is a compact form of visual information, with crowding present between different units of text (i.e., letters, words, and lines). Early evidence demonstrating the role of crowding in reading can be found in a line of psychophysical research on visual span arguing that the number of letters readers can recognize without making an eye movement (i.e., the visual span) serves as a low-level sensory limitation on reading (Legge et al., 2007). Consistent with this visual span hypothesis, these studies revealed that manipulations of text properties, including contrast, text size, text spacing, affected the size of reader’s visual span, which in turn highly correlated with their reading speed (Legge et al., 2001; Legge et al., 2007; Yu et al., 2007). For the purpose of understanding the limiting factor of visual span size, two studies dissociated visual acuity and visual crowding in separate psychophysical measurements and found that crowding, but not acuity, accounted for the observed correlation with reading speed (Levi et al., 2007; Pelli et al., 2007). These findings in turn led to the conclusion of reading speed being proportional to the reader’s crowding susceptibility.

It is important to note that although psychophysical studies provided compelling evidence to support the role of crowding in reading, these studies measured reading speed by means of rapid serial visual presentation (RSVP; Forster, 1970; Potter, 1984), thus making their findings not readily generalizable to reading in natural settings (Benedetto et al., 2015; Rayner et al., 2016). A key difference between RSVP and natural reading is that, in the latter scenario, eye movements are involved and therefore readers are required to select saccade targets at different levels of text. When reading a passage, readers are required to decide where to fixate within a word (McConkie et al., 1988; Rayner, 1979), which word to fixate next (Brysbaert & Vitu, 1998), and where to fixate on the following line (Hofmeister et al., 1999; Rayner, 1998). Additionally, RSVP differs from natural reading in the number of words processed at a given fixation. In reading research, it is well established that a reader’s word processing includes both the fixated word and the word in the region slightly right to the fixation point (i.e., parafoveal processing; see Schotter et al., 2012, for a review), as preventing information in parafovea largely slows down reading speed (McConkie & Rayner, 1975; Rayner, 2014). Conversely, as words are presented one at a time in RSVP paradigms, only foveal word processing is permitted whereas parafoveal processing is eliminated. Taken together, it is apparent that the current understanding of the role of crowding in reading is far from complete and eye-tracking studies, during which reading resembles natural reading to a higher extent, are needed to bridge this gap.

Cognitive processes associated with word recognition are often assumed to be the driving force behind eye movements during reading (Engbert et al., 2005; Reichle et al., 2003), thus we first review research on how crowding influences word recognition. To investigate crowding effects on word recognition, previous studies induced differences in crowding by manipulating the distance between letters (i.e., letter spacing; Eriksen & Eriksen, 1974) and recorded participant’s performance in isolated word recognition tasks (e.g., lexical decision task, naming task). For instance, Perea et al. (2011) presented words with subtly varied letter spacing and found that the reader’s response time decreased linearly as letter spacing increased. Later studies revealed that the facilitative effect of reduced crowding likely takes place during early perceptual encoding, as the facilitation was found to be independent of lexical factors (e.g., word frequency; Perea et al., 2011; Slattery et al., 2016). However, reducing crowding by means of increasing letter spacing does not yield unbounded benefits on word recognition (for discussions, see Slattery et al., 2016; van den Boer & Hakvoort, 2015). Specifically, increasing the amount of letter spacing over a certain criterion, likely around the width of a single character (Slattery et al., 2016), was shown to damage the perceptual integrity of words and hinder the reader’s parallel letter-recognition processing, which together outweighed benefits of reduced crowding and resulted in prolonged word recognition time (Cohen et al., 2008; Risko et al., 2011; Vinckier et al., 2011).

Reading research using eye-tracking methodology measures the time readers spend fixating on a word (i.e., fixation duration) as an indicator of the amount of cognitive effort associated with recognizing that word (Rayner, 1998, 2009). Take word frequency, a factor known to be influential to lexical processing difficulty, as an example whereby words of high frequency, such as “chair,” often receive shorter fixation durations than low-frequency words, such as “scone” (Inhoff & Rayner, 1986; Rayner & Duffy, 1986). Although crowding effects on fixation duration were indirectly obtained in several word-spacing studies (Drieghe et al., 2005a; Rayner et al., 1998), direct demonstrations were provided by later studies in which letter spacing manipulations were implemented. For instance, Slattery and Rayner (2013) induced subtle changes in letter spacing and found facilitative effects of reduced crowding manifested by words with wide letter spacing receiving shorter fixation duration compared to words with condensed letter spacing (see also Perea et al., 2016; Perea & Gomez, 2012). Further, the benefit of reduced crowding was only observed in early eye-movement measures that are indicative of early visual processing (Radach & Kennedy, 2013) and the effect was independent of lexical variables (e.g., word frequency; Li et al., 2018; Slattery & Rayner, 2013), which together suggested that the influence of crowding on word processing during reading was qualitatively similar to that when words were presented in isolation. However, these studies also discovered that the total time readers spent reading text materials (i.e., sentences, passages) was similar across letter spacing conditions despite early fixation durations being shorter when letters were widely spaced. This in turn suggested that whilst how crowding affects word recognition is well understood, research on the ways in which crowding influences additional components of natural reading (i.e., parafoveal processing, saccade targeting) is required to establish an understanding of the relationship between crowding and reading.

The extent to which crowding constrains parafoveal processing during reading is interesting for two reasons. First of all, according to Bouma’s law (Bouma, 1970, 1973), crowding is more damaging to object recognition in the parafovea than in the fovea due to higher eccentricity. This in turn predicts crowding as a stronger constraint on word recognition when words are presented away from the fovea, a prediction often corroborated in psychophysical studies (Chung, 2002, 2004) but not yet directly tested in eye-tracking experiments. Secondly, although crowding and visual acuity are often described by reading researchers as limiting factors on parafoveal processing (Schotter et al., 2012), quantitative models of eye-movement control during reading (i.e., E-Z Reader, SWIFT; Engbert et al., 2005; Reichle et al., 2003) uniformly formulate eccentricity-dependent drop in processing efficiency as a function of visual acuity alone, an assumption that lacks support from empirical evidence. Parafoveal processing in reading can be investigated by measuring parafoveal preview benefit and/or word skipping (Schotter et al., 2012). In a gaze-contingent display-change experiment (Rayner, 1975), an invisible boundary is positioned to the left of a target word and the information at the target location before the reader’s eyes cross the boundary (i.e., preview) is manipulated. Preview benefit – the difference in the reader’s fixation duration on the target word when it was preceded by either a valid preview (i.e., target word) or an invalid preview (e.g., visually dissimilar nonword) –can be calculated and compared across experimental conditions. Word skipping, a phenomenon referring to words occasionally being skipped (i.e., not fixated during first-pass) during reading, can also be used to measure parafoveal processing, as skipped words can only be processed parafoveally and are known to be processed to some extent before being skipped (Drieghe et al., 2005b).

The existing literature on how crowding affects parafoveal processing during reading is limited, with two lines of research providing only inconclusive evidence. The first line of research adopted the individual difference approach similar to psychophysical studies, with RSVP reading replaced by eye-tracking experiments implementing parafoveal preview manipulations. For instance, Risse (2014) measured a reader’s visual span (Legge et al., 2007) and their eye movements in a display-change reading experiment. In contrast to the robust correlations observed in psychophysical research (*r*^2^ > 0.95), this study found only weak correlations between crowding and reading speed and no reliable relationship between a reader’s crowding susceptibility and the magnitude of the reader’s parafoveal preview benefit effects, which suggested crowding played a negligible role in parafoveal processing (see also Frömer et al., 2015). However, it is important to note that the observed null findings may be a result of insufficient statistical power, as it requires substantially large samples to detect the predicted correlations in eye-tracking and reading experiments. Findings related to the effect of crowding on parafoveal processing can also be seen in letter spacing studies in which word skipping was reported (Korinth et al., 2020; Li et al., 2018). Specifically, these studies found reduced word skipping when reading widely spaced sentences and concluded that despite reduced crowding facilitating early encoding of the foveal word, larger letter spacing pushed letters further away from the fixation point and consequently hindered parafoveal processing. In other words, visual acuity rather than crowding served as the critical determinant of parafoveal processing. Nevertheless, the lower word skipping probability observed when letters were widely spaced does not necessarily negate the role of crowding in parafoveal processing, as low-level visual factors, such as a word’s physical length, were not equated across the comparison between words with wide and normal letter spacing. Studies presenting readers with words with an identical number of letters yet different in physical length have consistently found differences in word-skipping probability (Hautala et al., 2011; Hermena et al., 2017), suggesting a low-level influence on word skipping. As such, the reduced skipping of words with wide letter spacing may be reflective of a reader’s use of low spatial-frequency information rather than parafoveal word processing.

In addition to word recognition, reading involves deciding where to send the eyes, a process termed *saccade targeting* (for reviews, see Rayner, 1998, 2009). Whether crowding affects saccade targeting is of interest because whilst saccade targeting relies on spatial information located at different levels of eccentricity (e.g., next word, beginning of next line), the extent to which spatial information is preserved depends on the eccentricity of the intended saccade target. Saccade targeting at the sentence level involves readers deciding where to fixate on the next word (i.e., initial landing position). Previous studies consistently showed that the center of the distribution of the reader’s initial landing positions is at a location slightly left to the word center, a phenomenon called the preferred viewing location (PVL; Rayner, 1979) and has been interpreted as readers aiming for the word center (optimal viewing position; O'Regan & Jacobs, 1992; O'Regan et al., 1984) but falling short due to saccadic range errors (McConkie et al., 1988). For readers to implement such a saccade targeting strategy, low spatial-frequency information, such as word length and inter-word spaces, is crucial, as removing inter-word spaces was shown to eliminate the PVL phenomenon (Rayner et al., 1998). When reading multiline text, saccade targeting also includes return sweeps: saccades that bring the eyes from the end of one line to the beginning of the next line (Hofmeister et al., 1999; Rayner, 1998). Return sweeps are substantially longer than intra-line saccades and, as a result, more likely to undershoot the intended target due to stronger saccadic errors (McConkie et al., 1988). To study return sweep, undersweeps – the eyes making a leftward saccade immediately after return sweeps (Parker et al., 2017) – can be used as an indicator of return sweep accuracy. More specifically, recent studies demonstrated that undersweeps reflect oculomotor correction after undershooting the intended return sweep target (i.e., beginning of the next line) and its prevalence can be reduced by increasing saliency of the line-initial words (Slattery & Parker, 2019; Slattery & Vasilev, 2019).

The effect of crowding on saccade targeting depends on the decision at hand. When selecting targets for intra-line saccades (i.e., initial landing position), readers rely on spatial information located at on average approximately 8 characters to the right of the fixation point. However, when making inter-line saccades (i.e., return sweeps), targets are often more than 50 characters away from the fovea. Consistent with the idea that saccade targeting is different in these two situations, previous studies in which letter spacing was manipulated showed little or no difference in initial landing position when measured using percentage into the target word (Paterson & Jordan, 2010; Perea & Gomez, 2012; Slattery & Rayner, 2013). Conversely, the role of crowding on targeting return sweeps was demonstrated by earlier studies in which line spacing was manipulated (Kolers et al., 1981; Kubota, 1991; see Morrison & Inhoff, 1981, for a review). For instance, Kubota (1991) manipulated line spacing and found that undersweeps were less frequent when lines were more widely spaced. However, it is important to note that these findings were based on either coarse eye-movement measures or visual inspection of eye-movement patterns and therefore should be considered with caution.

In summary, it is clear from an examination of past research that despite a robust association between crowding and reading established in psychophysical research, the ways in which crowding constrains additional processes involved in natural reading, such as parafoveal processing and saccade targeting, remain largely unknown. The current study, as a crucial step towards bridging this gap, investigated crowding effects on reading via two eye-tracking experiments. Experiment 1 was a sentence-reading experiment in which letter spacing and preview validity were jointly manipulated. Critically, preview benefit effects are expected to be larger when crowding was reduced by means of wider letter spacing. Experiment 2 was a passage reading experiment in which line spacing was manipulated. This experiment allowed the test of crowding effects on word skipping whilst controlling physical width of the words and sentences and the examination of how crowding influences return sweeps. Increased word skipping and less undersweeps when crowding is reduced through wider line spacing is expected.

## Experiment 1

In Experiment 1, we conducted a sentence-reading experiment and manipulated the spacing between letters (wide, standard, and condensed letter spacing) and the validity of reader’s parafoveal information (valid, invalid parafoveal information). If crowding plays a role in parafoveal processing, we expect the difference in fixation duration between valid and invalid parafoveal information to be larger when letter spacing was wide (i.e., less crowding). Conversely, if crowding plays a negligible role in parafoveal processing, we expect the inversed pattern (i.e., smaller effect of preview validity as letter spacing increases) as wider letter spacing increases retinal eccentricity.

### Method

#### Participants

A total of 36 undergraduate students from the University of Southampton were recruited as participants. All participants were native English speakers, had no known reading difficulties, and had normal or corrected-to-normal vision. Participants received course credits in psychology courses as compensation for their participation. At this sample size, we were able to test an effect with 0.34 Cohen’s *d* at 0.5 statistical power. The sample size was determined based on previous letter spacing studies (Li et al., 2018; Slattery & Rayner, 2013) and the minimum number of participants required to counterbalance the experimental manipulation and the order in which the two experiments were completed (see *Procedure* section below).

#### Apparatus

Participants were seated 65 cm away from a 20-in. ViewSonic G225f monitor with a 75-Hz refresh rate. At this viewing distance, 1° visual angle was occupied by 2.38, 2.85, 3.56 characters in the wide, standard, and condensed letter spacing conditions, respectively. Sentences were written in 19-point Inconsolata font and displayed on a single line located at the middle of the screen. Participant’s eye movements were recorded using an EyeLink 1000 eye-tracker sampling at 1,000 Hz. In order to implement the gaze-contingent boundary technique (Rayner, 1975), an invisible boundary was positioned between the word prior to the target word and the space immediately preceding the target word. The display changes were triggered by three samples crossing the invisible boundary. Though participants were instructed to read binocularly, eye movements were only recorded from their right eye.

#### Materials and design

A total of 96 sentences were selected from three published studies (Drieghe et al., 2017; Fitzsimmons & Drieghe, 2011; White et al., 2016). The sentences were nine to 17 words in length (M = 12.94, SD = 1.54) with a target word positioned near the middle (Ordinal target word number: M = 6.2, SD = 1.03). All target words were five letters in length and not predictable from the preceding sentence context (see original studies for details of the material). According to the SUBTLEX-UK database (van Heuven et al., 2014), target words included low- to high-frequency words (Zipf frequency: M = 4.04, SD = 0.61, Min = 3.0, Max = 5.031).

**Fig. 2:**
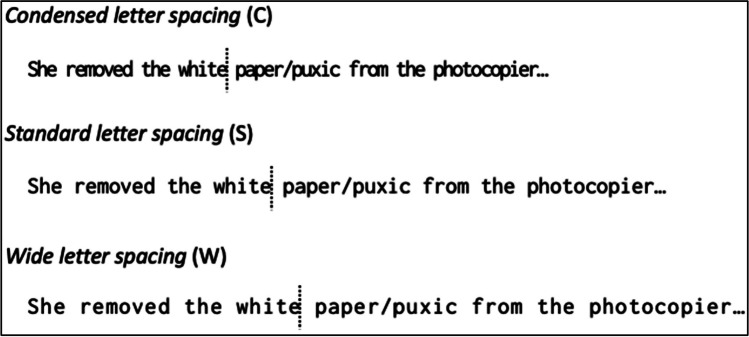
Example stimuli for Experiment 1. *Note*. Invisible boundaries used for boundary display change were marked with dotted lines. Valid previews are written before the slash, whereas invalid previews were written after the slash

Example stimuli are shown in Fig. 2. Letter spacing and preview validity were manipulated within participants. Letter spacing included three levels: Wide (W), standard (S), and condensed (C) letter spacing. Whilst default spacing of the Inconsolata font was used as the standard letter spacing condition, the wide and condensed letter spacing conditions were created by adding and subtracting 10% letter spacing, respectively. Average center-to-center distances between letters were 0.42, 0.35, and 0.28° visual angle for the wide, standard, and condensed letter spacing conditions, respectively. Preview validity included two levels: valid (V) and invalid (I) preview. Participants were presented with the target word itself (i.e., valid preview) or a pronounceable nonword (i.e., invalid preview) before their eyes crossing the invisible boundary. After crossing the boundary, participants saw the target word regardless of preview validity condition. The pronounceable nonwords were generated by keeping the first letter identical to the target and substituting the remaining four letters (*shico* as the preview of *stone*). Note that the invalid parafoveal preview and target words were both 5-letter in length. Overall, Experiment 1 had a 3 x 2 experimental design with 16 sentences assigned to each condition.

#### Procedure

Upon arriving for the experiment, participants were first given a consent form and instructions. After giving informed consent, participants were asked to rest on a chin rest to minimize head movements. A three-point calibration procedure was then carried out. Before reading each sentence in the experiment, a drift check was performed by asking participants to fixate on a fixation point shown at the left-center of the screen. If successfully completed, the fixation point was then replaced by the first letter of the sentence. Participants were instructed to silently read the sentences and were informed that a yes/no question would appear after one-third of the sentences. Prior to the experiment, participants read six practice sentences to familiarize themselves with the procedure. The experimental sentences were presented according to a pseudorandom order selected from six lists to counterbalance the experimental conditions across participants. Participants read a total of 102 sentences, which took approximately 20 minutes to complete. Note that participants also took part in Experiment 2. The order in which the two experiments were completed was counterbalanced across participants.

### Results

Average comprehension question accuracy was 93%, which indicated that participants understood the sentences correctly. Fixations shorter than 80 ms and within a three-character distance were merged together. Remaining fixations shorter than 80 ms or longer than 1,000 ms were removed. Trials in which (1) eye blink or track loss occurred whilst participants were fixating on the target word, (2) the target word received the first or last fixation in a given trial, (3) the fixation duration on the target word was more than 3 standard deviations away from the grand mean, and (4) when the display change occurred too early (i.e., triggered by fixations or saccades before the boundary) or too late (i.e., occurring more than 9 ms after boundary crossed) were removed. Overall, 84.9% of the data remained in the analysis.

The dependent measures included *First Fixation Duration* (FFD; duration of the first first-pass fixation on a word), *Single Fixation Duration* (SFD; duration of the fixation on a word when it was fixated exactly once during first-pass reading), *Gaze Duration* (total duration of all first-pass fixations made on a word), and *Total Viewing Time* (total duration of all fixations made on a word). *Skipping Probability* (Skip; probability of a word being skipped during first-pass reading), *Refixation Probability* (Refix; probability of a word receiving more than one first-pass fixations), and *Initial Landing Position* (ILP; position of the first first-pass fixation on a word) were also computed.

We constructed linear mixed-effect models (LMMs) for all dependent measures to provide inferential statistics. LMMs were constructed using the *lme4* (Version 1.1-29; Bates et al., 2014) and *lmerTest* (Version 3.1-3; Kuznetsova et al., 2017) packages in R version 4.1.2 (R core team, 2021). Generalized linear mixed-effect models were constructed with binomial distribution for binary measures (i.e., Skip, Refix). Letter spacing and preview validity were entered using successive differences contrast (Schad et al., 2020). Target word frequency was also entered as fixed effect in the word level analysis to account for its influence on the dependent variables. Following Barr et al.’s (2013) suggestion, we started from models with the maximal random effect structure and trimmed the models until convergence was achieved (see Appendix A for model trimming process). We also performed a sentence-level analysis, through which we found similar total sentence reading time across letter spacing conditions (see Appendix B), confirming that our letter spacing manipulation was subtle and not disruptive to the physical integrity of words (Paterson & Jordan, 2010).

**Fig. 3:**
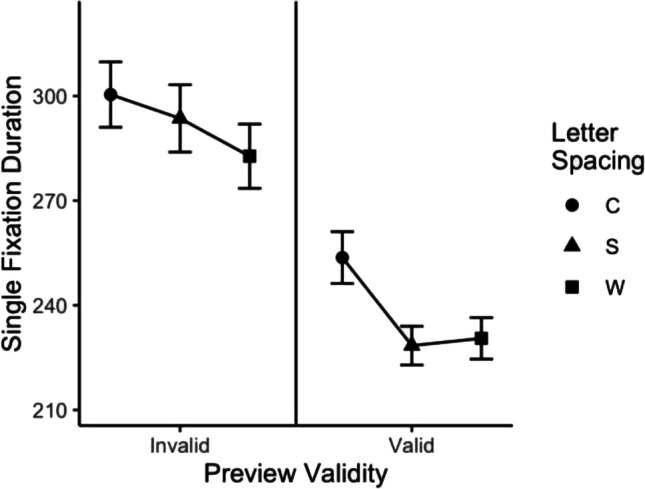
Mean single fixation duration in Experiment 1. *Note*. Error bars represent standard error of measure (SEM)

Descriptive statistics for the dependent measures are provided in Table 1. Statistics from (G)LMMs are provided in Table 2. Word frequency had significant effects on all fixation duration measures. Readers fixated longer on low frequency target words than on high-frequency target words. Parafoveal preview validity also had significant influences on all fixation duration measures. Readers spent longer fixating on target words when they were preceded by invalid previews compared to when the parafoveal preview was valid. Letter spacing effects on fixation duration measures were only reliable when comparing between the standard and condensed letter spacing conditions. Target words received longer fixation durations when letter spacing was condensed than when letters were of standard letter spacing. Finally, there were (marginally) significant interactions between preview validity and letter spacing on single fixation duration, indicating that the difference between valid and invalid preview was largest in the standard letter spacing condition and decreased in magnitude when letter spacing was increased or decreased (see Fig. 3). Follow-up contrasts revealed that (1) the condensed letter spacing condition had a longer single fixation duration than the standard letter spacing condition only when participants had intact parafoveal preview of the target words (*t*_Valid_ = -4.33, *p* < .001; *t*_Invalid_ = -1.11, *p* = .267) and (2) the wide letter-spacing condition had a slightly shorter single fixation duration when parafoveal preview was invalid (*t*_Valid_ = 0.91, *p* = .364; *t*_Invalid_ = -1.69, *p* = .092). Other early fixation duration measures (i.e., first fixation duration and gaze duration) showed qualitatively similar trends.

**Table 1:** Descriptive statistics for dependent measures in Experiment 1

Letter spacing	Preview validity	FFD	SFD	GD	TVT	Skip	Refix	ILP
Condensed	Valid	248.21 (6.84)	253.68 (7.43)	274.86 (9.16)	322.31 (10.51)	16.17 (2.34)	10.31 (1.87)	0.47 (0.02)
	Invalid	285.02 (7.98)	300.42 (9.37)	335.05 (9.78)	396.71 (13.49)	9.65 (2.03)	19.68 (2.43)	0.42 (0.02)
Standard	Valid	228.23 (4.91)	228.44 (5.55)	252.9 (6.73)	295.2 (10.01)	12.36 (1.91)	12.2 (1.94)	0.45 (0.02)
	Invalid	278.57 (8.39)	293.58 (9.65)	327.64 (10.92)	382.58 (14.01)	8.13 (1.62)	19.43 (3.34)	0.44 (0.01)
Wide	Valid	227.12 (5.29)	230.52 (5.95)	262.3 (7.52)	301.71 (10.91)	6.96 (0.93)	17.33 (2.91)	0.45 (0.02)
	Invalid	268.59 (8.28)	282.74 (9.22)	323.16 (10.4)	382.62 (13.13)	3.25 (0.9)	25.4 (2.95)	0.46 (0.02)

Standard errors are provided in parentheses. Fixation duration measures were measured in milliseconds. ILP was measured as proportion into words

**Table 2:** (G)LMM results for dependent measures in Experiment 1

Fixed effect	Statistics	FFD	SFD	GD	TVT	Skip	Refix	ILP
Intercept	Estimate	5.49	5.52	5.61	5.75	-2.63	-1.87	0.45
	Std. Error	0.02	0.02	0.03	0.03	0.15	0.17	0.01
	t/z-value	**238.43**	**229.02**	**222.61**	**202.2**	**-18.03**	**-11.27**	**37.04**
Word frequency	Estimate	-0.05	-0.06	-0.08	-0.09	0.23	-0.33	–^a^
	Std. Error	0.01	0.01	0.01	0.02	0.13	0.1	–
	t/z-value	**-4.25**	**-4.87**	**-5.46**	**-4.97**	1.73	**-3.47**	–
	*p*-value	**<.001**	**<.001**	**<.001**	**<.001**	.084	**<.001**	–
Preview (V – I)	Estimate	-0.16	-0.2	-0.24	-0.26	0.7	-0.7	0.02
	Std. Error	0.02	0.02	0.02	0.02	0.15	0.11	0.01
	t/z-value	**-7.95**	**-9.06**	**-13.73**	**-12.89**	**4.79**	**-6.45**	1.73
	*p*-value	**<.001**	**<.001**	**<.001**	**<.001**	**<.001**	**<.001**	.084
Space (S – C)	Estimate	-0.05	-0.06	-0.06	-0.06	-0.29	0.12	-0.01
	Std. Error	0.01	0.02	0.02	0.02	0.15	0.14	0.01
	t/z-value	**-3.44**	**-3.77**	**-3.47**	**-3.44**	*-1.89*	0.85	-0.53
	*p*-value	**<.001**	**<.001**	**<.001**	**<.001**	*.059*	.398	.599
Space (W – S)	Estimate	-0.02	-0.01	0.01	0.02	-0.81	0.38	0.01
	Std. Error	0.01	0.02	0.02	0.02	0.19	0.13	0.01
	t/z-value	-1.35	-0.6	0.9	1.02	**-4.24**	**2.98**	1.14
	*p*-value	.176	.549	.368	.307	**<.001**	**.003**	.255
Preview x Space (S – C)	Estimate	-0.05	-0.06	-0.04	-0.03	-0.18	0.27	-0.03
	Std. Error	0.03	0.03	0.03	0.04	0.30	0.27	0.02
	t/z-value	-1.58	**-2.09**	-1.22	-0.68	-0.61	0.99	-1.25
	*p*-value	.114	**.037**	.222	.498	.541	.32	.213
Preview x Space (W – S)	Estimate	0.03	0.06	0.04	0.02	0.35	0.01	-0.02
	Std. Error	0.03	0.03	0.03	0.04	0.38	0.25	0.02
	t/z-value	1.08	*1.84*	1.39	0.55	0.92	0.05	-1.01
	*p*-value	.281	*.065*	.165	.584	.359	.963	.314

Fixation duration measures were log-transformed. Significant effects are indicated in **bold**. Marginally significant effects are indicated in *italics*

^a^Word frequency was not included as fixed effect for the ILP analysis as it has been shown to have little influence on ILP (Rayner et al., 1996)

**Fig. 4:**
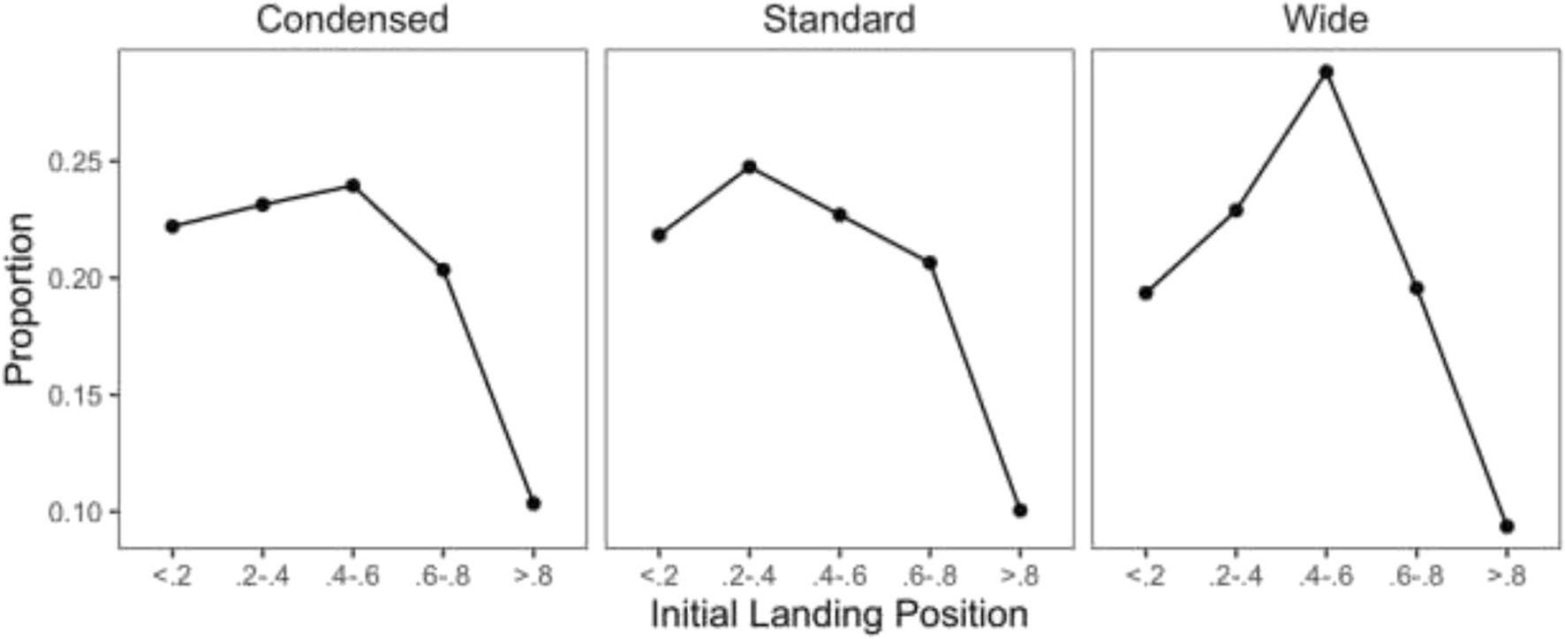
Initial landing position curves across letter spacing conditions in Experiment 1

Word frequency had significant influences on refixation probability. Participants were more likely to refixate low frequency target words than high-frequency target words. Preview validity effects were significant on skipping and refixation probability. Readers were more likely to skip and less likely to refixate on target words when they received valid preview of the target word than when preview was invalid. Letter-spacing effects on skipping and refixation probability were only significant when comparing between wide and standard letter-spacing conditions. Target words with wide letter spacing had lower skipping and higher refixation probability than target words with standard letter spacing. No significant two-way interactions were found. Preview validity and letter spacing had no influence on initial landing positions. Initial landing position curves (Fig. 4) showed that reader’s initial landing positions centered around word center regardless of the letter spacing manipulations.

### Discussion

Results of Experiment 1 revealed two interesting findings. First of all, increasing and decreasing letter spacing relative to standard letter spacing yielded qualitatively distinct effects on reader’s eye-movement behaviors. Whilst decreasing letter spacing resulted in inflated fixation duration, effects of increasing letter spacing were mainly observed on fixation probability measures (i.e., skipping and refixation probability). Secondly, interactions between letter spacing and preview validity indicated that crowding played a role in parafoveal processing. This was evidenced by elevated crowding between letters with condensed spacing reducing reader’s use of parafoveal information.

The effects of condensed letter spacing on fixation duration measures were consistent with those reported in previous studies (Korinth et al., 2020; Li et al., 2018). As condensed letter spacing improved visual acuity of letters within the fixated word, a prediction based solely on visual acuity would expect shorter fixation durations in the condensed letter spacing condition, a pattern opposite to the observed effects. The observed effect therefore supports the notion that visual acuity alone is insufficient to account for the low-level constraints readers encounter in word recognition during reading. Alternatively, as argued by earlier psychophysics research (Legge et al., 2007; Levi et al., 2007; Pelli et al., 2007), crowding likely plays a crucial role and, when the degree of crowding becomes stronger in the case of decreased letter spacing, imposes a limitation on visual processing and consequently slows down word recognition. Additionally, it is worth noting that the condensed letter spacing effects were present on the earliest fixation duration measure (i.e., first fixation duration) and remained at an approximately similar magnitude in later measures, indicating an early temporal locus that is in line with the sensory nature of crowding. Altogether, the observed condensed letter-spacing effects likely reflected crowding as a low-level constraint on foveal word processing during reading, in turn extending previous findings in RSVP paradigms (Chung, 2002; Legge et al., 2001; Legge et al., 2007) into reading in natural settings.

With regards to the influences of increased letter spacing, current results failed to replicate the previously observed facilitation on early fixation duration measures (Perea & Gomez, 2012; Slattery & Rayner, 2013). One possible explanation concerns the inevitable trade-off between visual acuity and crowding when letter spacing was manipulated. More precisely, as increasing letter spacing results in reduced crowding and visual acuity, an “optimal” letter spacing for word recognition may exist, possibly somewhere in between the standard and wide letter spacing conditions. When letter spacing was increased beyond this optimal point, the benefit of reduced crowding no longer outweighs the aggravated acuity degradation for letters close to the end of the fixated word, consequently forcing readers to program an additional inter-word saccade (i.e., refixation) to complete word recognition. Nevertheless, based on the results at hand we cannot rule out an alternative hypothesis in which perceptual constraints are assumed to play a minimal role in word recognition. Considering that saccade target selection, including word skipping and refixation, are known to be affected by a word’s physical length (Hautala et al., 2011; Hermena et al., 2017), effects of increased letter spacing on fixation probability measures may be caused by differences in a target word’s physical length across letter-spacing conditions: Target words with wide letter spacing were physically longer, which in turn reduced the probability of being skipped and increased the probability of receiving more than one fixation.

Following the trade-off explanation, the current results can be interpreted as a weak version of the increased letter spacing effects previously observed (Perea & Gomez, 2012; Slattery & Rayner, 2013). The increased probability of refixation reflects the reader’s response to the wide letter-spacing condition’s deviation from the optimal letter spacing and, when the degree of deviation becomes stronger, may lead to the previously observed letter spacing effects. Alternatively, based on the saccade targeting explanation, effects of increased letter spacing on word skipping and refixation together reflect reader’s use of low spatial-frequency information in parafoveal vision. At this point, we remain agnostic as to which of the explanations accounts for the observed results. It is also important to note that the two explanations need not to be mutually exclusive. Future research crossing word length with letter spacing manipulations (Korinth et al., 2020) and/or implementing computational models in which acuity and crowding are simultaneously formulated will be required to ascertain the boundary conditions or relative contributions of both possibilities to letter spacing effects on eye-movement behaviors.

The finding of crowding effects on single fixation duration qualified by validity of parafoveal information confirmed the main hypothesis and supported the role of crowding in parafoveal processing during reading. One way to interpret the observed interaction was that when words were presented in the reader’s parafoveal vision, where crowding was stronger due to higher eccentricity (Bouma, 1970, 1973), elevated crowding due to condensed letter spacing caused a disruption on word recognition. However, when parafoveal information was prevented and words were only subsequently processed with foveal vision, where crowding was found to be trivial (Liu & Arditi, 2000; Toet & Levi, 1992) or non-existent (Strasburger et al., 1991), differences in levels of crowding no longer exerted influence on word processing. On one hand, a strong version of this interpretation would contend that crowding only affects parafoveal word processing during reading and argue that the letter-spacing effects observed in previous studies (Korinth et al., 2020; Li et al., 2018) were lag effects (Radach & Heller, 2000; Vitu et al., 2001) in disguise. Specifically, as word processing in natural reading commences in the reader’s parafoveal vision, the previously observed crowding effects on fixation duration can be caused by differences only in parafoveal processing of the target words but not subsequent foveal processing when directly fixated. By contrast, one may maintain a role of crowding in foveal word processing by arguing that the current results were caused by differences in foveal load affecting reader’s parafoveal processing (Henderson & Ferreira, 1990). More precisely, as letter spacing was manipulated at the sentence level, it is possible for crowding to disrupt foveal recognition of the word prior to the target and consequently reduce the amount of time available for processing the target word in parafoveal vision. However, this interpretation is less likely to hold considering that models of reading (Reichle et al., 2003) assumed only lexical variables but not low-level sensory factors, such as crowding, affect parafoveal processing of the upcoming word (see Drieghe, 2008, for a discussion). Finally, it is important to acknowledge the fact that the interaction only emerged on one fixation duration measure, suggesting that this finding should be considered cautiously and future replications are needed.

Additionally, there was a marginal significant interaction between preview validity and the contrast between wide and standard letter spacing conditions on single fixation duration indicating reduced use of parafoveal information when letter spacing was increased. One way of understanding this pattern was that words with wide letter spacing had a slight benefit over those with standard spacing when parafoveal preview was invalid (i.e., a pronounceable nonword), which in turn raises the issue of how preview masks used in the invalid preview condition were processed. Recent studies showed that preview masks commonly used in display change experiments (e.g., Xs, random letter strings, nonwords) were not representative of a neutral baseline but rather an interference (Hutzler et al., 2013; Kliegl et al., 2013; Vasilev & Angele, 2017; Yan et al., 2012) that increases as preview duration increases (Pan et al., 2020). As an alternative, parafoveal degradation, achieved via randomly omitting black pixels from the parafoveal word, was proposed since such an approach reduced the interference caused by presenting preview masks and therefore served as a cleaner baseline for the calculation of preview benefit effects (Gagl et al., 2014; Marx et al., 2015; Vasilev et al., 2018). Provided that wide letter spacing pushing the parafoveal word further away results in added perceptual constraint on parafoveal processing, the observed interaction can be construed as wide letter spacing, resembling the influences of parafoveal degradation, reducing the interference associated with the preview mask. Questions regarding why increasing and decreasing letter spacing exerted influences on fixation duration differently according to the availability of parafoveal information requires future investigation.

## Experiment 2

In Experiment 2, we conducted a passage-reading experiment and varied the amount of spacing between lines (wide, standard, condensed line spacing). If vertical crowding between lines affects foveal and parafoveal processing, we expect shorter fixation duration and higher skipping probability when line spacing was increased (i.e., reduced crowding). Additionally, if crowding influences inter-line saccade targeting, we expect more accurate return sweeps and therefore less undersweeps when line spacing was increased.

### Method

#### Participants

The 36 participants from Experiment 1 also participated in Experiment 2.

#### Apparatus

The apparatus was identical to that in Experiment 1. Four-line passages were written in 19-point Inconsolata font, left justified, and positioned at screen center. At 65-cm viewing distance, 2.85 characters occupied 1° visual angle.

#### Materials and design

**Fig. 5:**
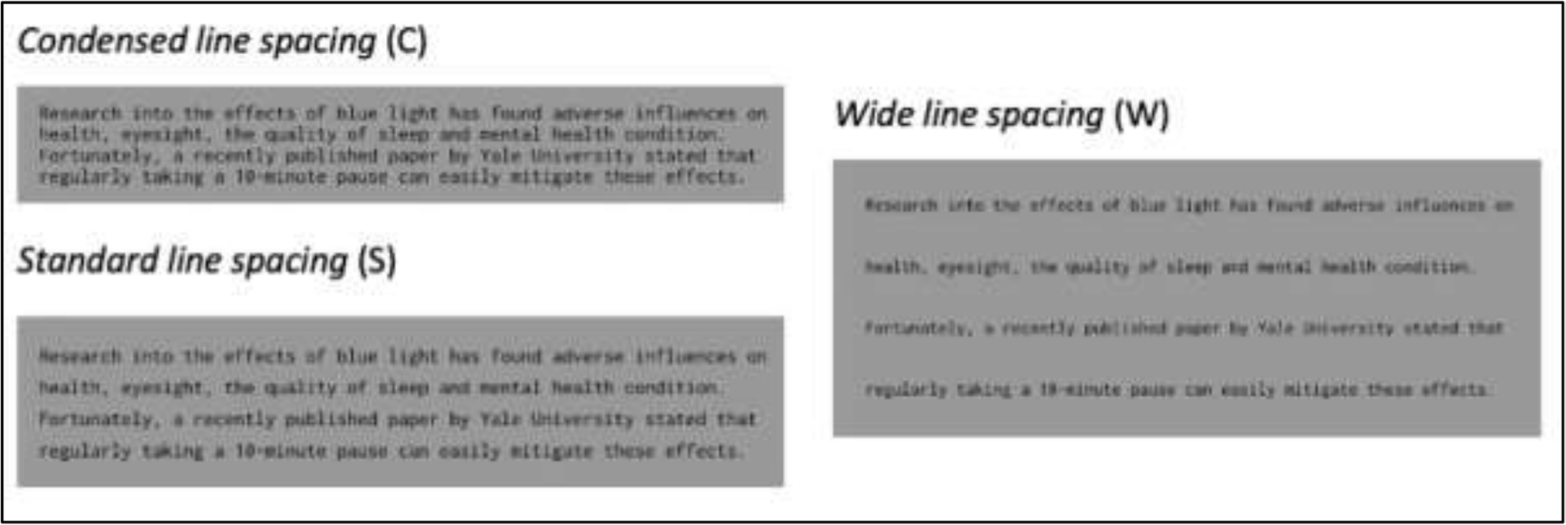
Example stimuli for Experiment 2

A total of 60 passages were excerpted from UK national newspapers, sampled from a wide variety of topics. Passages were 42–67 words in length (M = 52.4, SD = 4.92) and separated into four lines (number of words in line: M = 13.09, SD = 1.68). Excluding words (1) with upper case letters, (2) included Arabic numbers, (3) included or located next to punctuation marks, (4) at the beginning or ending of lines, and (5) function words, a total of 1,102 words were entered in the analysis. Word length ranged from two to 13 letters (M = 5.62, SD = 1.9), whereas Zipf word frequency (van Heuven et al., 2014) ranged from 1.3 to 7.18 (M = 4.84, SD = 0.92). Example stimuli are provided in Fig. 5. Line spacing was manipulated within participants and included three levels: Wide (W), standard (S), and condensed (C) line spacing. Line spacing was 1.75, 0.45, and 0.09° visual angle for the wide, standard, and condensed line-spacing conditions, respectively. Overall, Experiment 2 had a 1 x 3 within-participant design, with 20 passages allocated to each line spacing condition.

#### Procedure

Procedure was similar to Experiment 1 with the following exceptions. Participants underwent a 9-point calibration, read three practice passages followed by 60 experimental passages, which together took approximately 30 minutes to complete. The passages were presented based on a pseudorandom order selected from three lists to counterbalance the line spacing manipulation across participants.

### Results

Responses to comprehension questions had an average of 83%, which suggested that readers understood the passages correctly. Data cleaning was conducted according to the same standard as Experiment 1 (but excluding criteria concerning display changes), resulting in 97.2% of data entering the analysis. All dependent measures except for initial landing position were calculated for every word. Additionally, Undersweep Probability (US; probability of readers making a leftward saccade immediately after making a return sweep) was calculated. LMMs were constructed following the same procedure as in Experiment 1 (see Appendix C for model trimming process). Line spacing was coded using successive differences contrast. Line spacing, word length, and word frequency were entered as fixed effects, whereas word and participant identifiers were treated as random effects.

Descriptive statistics for dependent measures are summarized in Table 3. Results from the (G)LMMs are summarized in Table 4. Word length had significant effects on gaze duration, total viewing time, skipping probability, and refixation probability. Participants spent longer fixating, and were less likely to skip and more likely to refixate long words when compared to short words. Word-frequency effects were significant on all measures. Low-frequency words were fixated longer, were less likely to be skipped, and more likely to be refixated compared to high-frequency words. Increasing line spacing from standard to wide had significant influences on fixation duration measures. Readers had shorter fixation durations when fixating on words that had wider line spacing. Line spacing also had significant influences on skipping probability. This effect was significant across all levels of line spacing and manifested by readers more likely to skip words when line spacing was increased. The effect of line spacing on refixation probability was only significant between standard and wide line spacing. Words that were further away from adjacent line(s) were less likely to be refixated compared to those with standard line spacing. Finally, a significant difference in undersweep probability was obtained. Readers were more likely to make a leftward corrective saccade after return sweeps when line spacing was increased.

**Table 3:** Descriptive statistics for dependent measures in Experiment 2

Line spacing	FFD	SFD	GD	TVT	Skip	Refix	US
Condensed	232.31 (5.67)	232.83 (5.89)	260.31 (6.65)	311.44 (8.72)	22.31 (1.34)	11.11 (0.89)	36.48 (3.1)
Standard	230.11 (5.38)	230.69 (5.66)	260.12 (6.71)	309.17 (8.65)	23.49 (1.49)	11.78 (1.03)	39.78 (3.31)
Wide	227.74 (5.51)	228.15 (5.64)	256.27 (7.01)	305.55 (8.6)	25.33 (1.49)	10.79 (0.94)	41.24 (3.62)

Standard errors are provided in parentheses. Units for fixation duration measures are milliseconds

**Table 4:** (G)LMMs results for dependent measures in Experiment 2

Fixed effect	Statistics	FFD	SFD	GD	TVT	Skip	Refix	US
Intercept	Estimate	5.38	5.38	5.47	5.6	-1.46	-2.46	-0.57
	Std. Error	0.02	0.02	0.02	0.03	0.09	0.13	0.18
	t/z-value	**245.47**	**237.79**	**227.34**	**219.36**	**-15.68**	**-18.7**	**-3.12**
Word length	Estimate	0	0	0.02	0.03	-0.44	0.26	–
	Std. Error	0	0	0	0	0.01	0.01	–
	t/z-value	-0.15	-0.11	**6.99**	**10.74**	**-30.92**	**17.77**	–
	*p*-value	.879	.9	**<.001**	**<.001**	**<.001**	**<.001**	–
Word frequency	Estimate	-0.02	-0.02	-0.02	-0.02	0.14	-0.14	–
	Std. Error	0	0.01	0.01	0.01	0.03	0.03	–
	t/z-value	**-3.62**	**-3.1**	**-4.16**	**-2.89**	**5.27**	**-4.21**	–
	*p*-value	**<.001**	**.002**	**<.001**	**.004**	**<.001**	**<.001**	–
Space (S – C)	Estimate	-0.01	-0.01	0	0	0.08	0.07	0.17
	Std. Error	0	0	0.01	0.01	0.03	0.04	0.07
	t/z-value	**-2**	*-1.89*	-0.74	-0.49	**2.47**	1.59	**2.57**
	*p*-value	**.046**	*.059*	.461	.627	**.014**	.111	**.01**
Space (W – S)	Estimate	-0.01	-0.01	-0.01	-0.01	0.1	-0.11	0.07
	Std. Error	0	0	0.01	0.01	0.03	0.04	0.07
	t/z-value	**-2.15**	**-2.03**	**-2.39**	-1.34	**3.28**	**-2.55**	1.08
	*p*-value	**.032**	**.043**	**.017**	.187	**.001**	**.011**	.279

Fixation duration measures were log-transformed. Significant effects are indicated in **bold**. Marginally significant effects are indicated in *italics*

### Discussion

In Experiment 2, results were expected to show that reducing vertical visual crowding by means of increased line spacing facilitates passage reading behaviors. Consistent with the expectations, results showed benefits of increased line spacing on fixation duration and probability measures: Readers spent a shorter time fixating on and were more likely to skip words when line spacing was increased. However, in contrast to the expectation, results on undersweep probability failed to result in improvements on a reader’s return sweep accuracy. Readers were less accurate in making return sweeps, as more undersweeps occurred when line spacing was made wider.

The finding of increased line spacing reducing a reader’s fixation duration has two important implications. First, the observed effect extended previous line spacing research utilizing global reading time as dependent measure (Chan & Lee, 2005; Kruk & Muter, 1984; Van Overschelde & Healy, 2005) to the fixation times on individual words. The observed effects also improve understanding of cross-line interference during passage reading. In Pollatsek et al. (1993)’s gaze-contingent moving-window experiment, they found that the presentation of visually dissimilar words at the line below the current fixation interfered with the reading process and resulted in longer reading times. The results reported in the current study demonstrated that, in addition to the content from the line below, the spatial distance at which the neighboring lines are positioned affects the reading process. As vertical rather than horizontal spacing was manipulated, visual acuity of words within a line was controlled across line spacing conditions. Also, considering that word length and word frequency were included in the LMMs as fixed effects and passages were assigned to each line spacing condition equally often across participants, the observed effect could not be attributed to higher level factors. Taken together, it can be inferred that increasing the amount of spacing between lines reduced levels of vertical crowding, which in turn led to a facilitation on early visual processing of words. Further, although the differences between line spacing conditions were subtle, with increased line spacing saving merely 2–3 ms per word, the effect was present whilst the task involved reading passages and may accumulate into larger benefits when reading larger text bodies (e.g., books, novels).

Critical to the research aim was the observation of increased line spacing leading to increased word skipping. The magnitude of such effect was approximately 3% when comparing between the condensed and wide line spacing conditions, not that unsimilar to those of word frequency yet far smaller than that of word length (Brysbaert & Vitu, 1998). Considering that the physical length of words was controlled when making comparisons across line spacing conditions, influences of low spatial frequency information on word skipping (Hautala et al., 2011; Hermena et al., 2017) could not account for the observed effects. One interpretation concerns differences in the efficiency of parafoveal word processing. More precisely, as words have been found to be processed to a certain extent prior to being skipped (Drieghe et al., 2005b), line spacing effect on word skipping may reflect wider spacing between lines reducing levels of vertical crowding, which in turn facilitated reader’s early visual processing in parafoveal vision. Moreover, through comparing line-spacing effects on fixation duration and word skipping, there is a suggestion of the decision of whether to skip a word being more sensitive to variations in line spacing, as differences in skipping probability were statistically significant between all levels of line spacing. This feature, though merely based on qualitative comparison, suggested that line spacing had stronger effects on parafoveal processing than on foveal processing, in turn aligning with the known characteristic of crowding being influential in parafoveal vision (Bouma, 1970, 1973) and less pronounced in foveal vision (Liu & Arditi, 2000; Strasburger et al., 1991; Toet & Levi, 1992).

Conversely, as line spacing was manipulated at the passage level, observed line spacing effects on word skipping could also be interpreted in terms of global reading strategy (Brysbaert & Vitu, 1998; Radach et al., 2008). When readers were presented with widely spaced passages in which interferences from adjacent lines were reduced, they were more likely to adopt a “risky” reading strategy (O’Regan, 1990, 1992) and consequently make longer saccades and skip words more often. This possibility could be further bolstered when aspects of the experimental methodology were taken into account. Since passages across line-spacing conditions differed in total height but were uniformly centered vertically on the screen, the fixation point used during drift check, aligned to the first letter of the upcoming passage, were positioned differently on the vertical axis which in turn can be used as a pre-cue for the upcoming text configuration. For instance, if readers saw a fixation point further away from the vertical center point of the screen during drift check (i.e., higher along the vertical axis), they could predict that the upcoming passage has wide line spacing. Also, considering that the completion of the drift check was at least partially controlled by readers (i.e., deliberately looking away from the fixation point prevents trial onset), the drift check procedure could be used as a temporal buffer for readers to fine-tune their global reading strategy. This interpretation, albeit plausible, should be considered with caution due to the lack of evidence supporting global modulation on reading strategy in the literature.

Finally, contrary to previous findings, increasing line spacing diminished the reader’s return sweep accuracy and increased their probability of making corrective saccades after return sweeps. As the observed effect was based on statistical tests on an eye-movement measures more directly reflective of return sweep accuracy (i.e., undersweep probability; Parker et al., 2017), the current results are favored over those reported in previous line spacing studies (Kolers et al., 1981; Kubota, 1991) and indicate that crowding had no influence on return sweep accuracy. Alternatively, saccadic range error (McConkie et al., 1988), the phenomenon of readers being more likely to undershoot the intended saccade target when it was further away, likely played a role in the saccade target selection of return sweeps. Since increasing line spacing inevitably pushed line-initial words, the intended targets of return sweeps, further away, such manipulation forced readers to plan a longer return sweep saccade and therefore more likely undershoot due to oculomotor errors.

## General discussion

The current study was set out to investigate the role of visual crowding in parafoveal processing and saccade targeting during reading. In two eye-tracking experiments, we found that crowding affected measures of parafoveal processing, including parafoveal preview benefit and word skipping, but not other indicators of saccade targeting, such as initial landing position and return sweep. Collectively, these results suggest that whilst crowding constraints the efficiency of linguistic processing in parafoveal vision, it exerts negligible influence on saccade targeting within words and across lines during reading. We begin by discussing the theoretical implications of the role of crowding in foveal and parafoveal processing during natural reading, specifically the ways in which the current results relate to previous RSVP research (Chung, 2002, 2004; Legge et al., 2007) and models of eye-movement control during reading (Engbert et al., 2005; Reichle et al., 2003). We then discuss the applied values of the current findings in terms of designing reading aids targeting specific clinical populations.

The first issue concerns a comparison between the current findings and previous RSVP research (Chung, 2002, 2004; Legge et al., 2007). In general, the current results supported the notion of crowding as an early sensory limitation on reading (Legge et al., 2007; Levi et al., 2007; Pelli et al., 2007) and further extends the idea into a reading task of higher ecological validity (i.e., natural reading). The role of crowding in reading was not only preserved when eye movements were involved (see also Yu et al., 2007), but also extended to the processing of words aside from the fixated word. Nonetheless, one must acknowledge that albeit being statistically significant, the crowding effects obtained in natural reading tasks (Korinth et al., 2020; Li et al., 2018) were less pronounced when comparing to those reported in RSVP studies (Legge et al., 2007). One likely reason for the observed difference in magnitude lies in the ways in which these reading tasks are implemented. On the one hand, RSVP paradigms implemented in previous studies instructed readers to read words aloud as fast as possible, with no additional constraints on whether the order of words within the sentences were reported correctly or if the sentences were understood correctly. As such, the task emphasized the rate of early, sub-lexical processing (i.e., phonological processing) and likely accentuated the contribution of early sensory factors, such as crowding. Conversely, natural reading experiments instruct readers to read for comprehension and occasionally test readers with comprehension questions during the experiments. This in turn increases reader’s emphasis on higher-level processing (i.e., semantic, syntactic, discourse) and likely attenuates the influences of low-level, visual factors. Overall, the current results provide supporting evidence for crowding as an essential yet subtle constraint on reading in natural settings.

For the second issue, the ways in which the current findings shape models of eye-movement control during reading are discussed. First, it is important to note that in two experiments, crowding was found to exert influences on parafoveal preview benefit as well as word skipping. Although preview benefit and skipping can be categorized as indicators of parafoveal processing efficiency at face value, a closer examination of the literature indicated that the two measures differ in the extent of parafoveal processing involved (Drieghe et al., 2005b). More precisely, whilst preview benefit reflects the difference in parafoveal processing prior to reader’s eyes making the saccade onto the parafoveal word, the decision of word skipping must be made relatively early in time and can be made based on partial lexical recognition (Drieghe et al., 2005b; Rayner et al., 2011). As such, results of crowding influencing both preview benefit and skipping suggested a temporal locus of crowding effect on parafoveal processing commencing early in time. Taken together with the lack of interaction between crowding and lexical variables (i.e., word frequency; Li et al., 2018; Perea & Gomez, 2012; Slattery & Rayner, 2013) previously reported, the current findings place a temporal constraint on the influence of crowding on early visual processing in the parafovea. Furthermore, the demonstration of crowding as a sensory limitation on word identification in addition to retinal acuity sheds light on the formulation of visual constraints in models of eye-movement control during reading. At present, prominent models of reading (i.e., E-Z Reader, SWIFT; Engbert et al., 2005 ; Reichle et al., 2003) uniformly assume acuity degradation as the sole visual constraint on word identification and modelled word processing to decrease as a function of eccentricity (i.e., letters further away from fovea were processed less efficiently). The current results of crowding affecting word processing in fovea and parafovea in turn challenge these assumptions and argue that distance to nearby objects, the crucial determinant of crowding, should be taken into account whilst modelling word processing in reading (see also Veldre et al., 2022).

Finally, the applied value of the current findings is discussed. Although the magnitude of crowding effects obtained in the current study was subtle when tested on skilled adult reader’s eye-movement behaviors during reading, these effects may be stronger for readers from other populations, particularly those who were found to be more susceptible to visual constraints. For instance, in a sentence reading experiment, Li et al. (2018) found that elder readers were more disrupted when letter spacing was condensed when compared to young readers. Readers suffering from amblyopic vision (Levi et al., 2007) and a sub-population of dyslexic readers (Joo et al., 2018) were also found to be more strongly constrained by crowding during reading. With these population-specific findings in mind, the current results can in turn serve as a powerful tool for designing a battery of quantitative measurements that can be used to assess readers with visual impairments. The derived measures can be useful in assessing the specific components of natural reading that are limited by declined visual ability (e.g., reduced foveal/parafoveal processing, less accurate saccade targeting), potentially informing clinicians’ selection of the corresponding intervention plans and aiding graphic designers in designing text interfaces that are optimal for different reader populations. Future research contrasting the role of crowding in natural reading across populations will be fruitful for understanding the reading difficulties encountered by specific reader populations and guiding text designs aiming to ameliorate such difficulties.

In summary, the two experiments reported here provided compelling evidence for the role of crowding in eye movements during reading. Crowding, when manipulated via text spacing, influenced not only the recognition of the fixated word but also constrained parafoveal word processing. These findings not only extend the notion of crowding as a sensory limitation on reading (Legge et al., 2007; Pelli et al., 2007) into the online linguistic processing spanned across fovea and parafovea, but also shed light on the description of early visual processing in models of eye-movement control during reading (Engbert et al., 2005; Reichle et al., 2003). Future research will need to concentrate on developing a method of characterizing crowding effect during natural reading and on examining crowding effects across reader populations.

## Figures and Tables

**Fig. 6:**
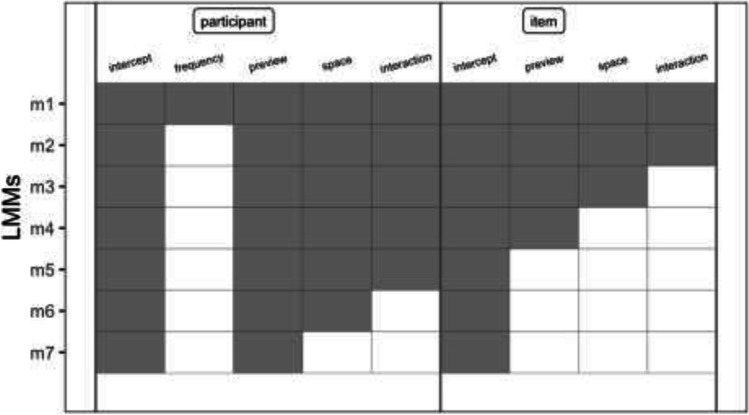
Model trimming for first fixation duration

**Fig. 7:**
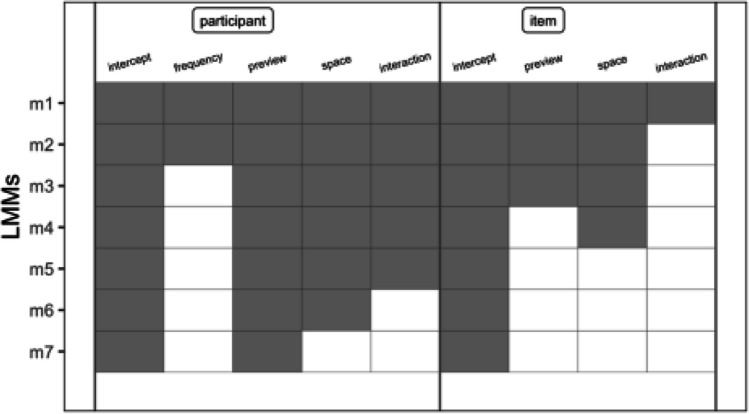
Model trimming for single fixation duration

**Fig. 8:**
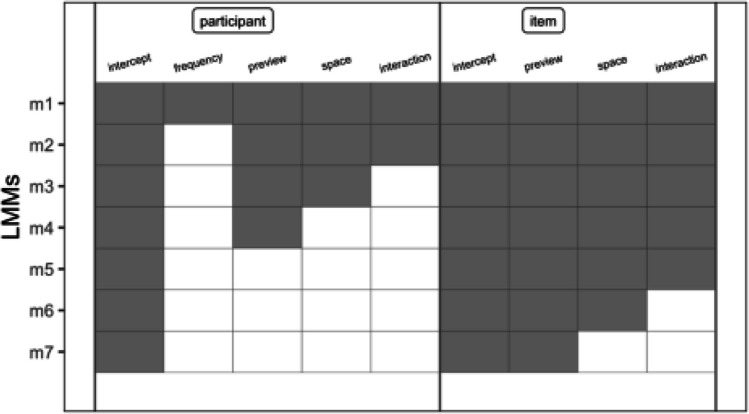
Model trimming for gaze duration

**Fig. 9:**
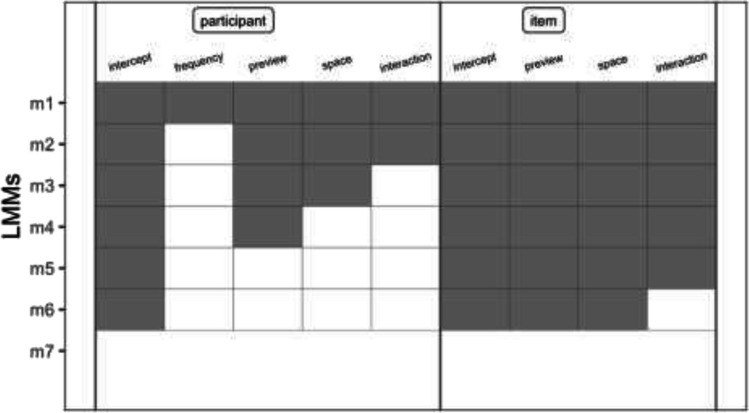
Model trimming for total viewing time

**Fig. 10:**
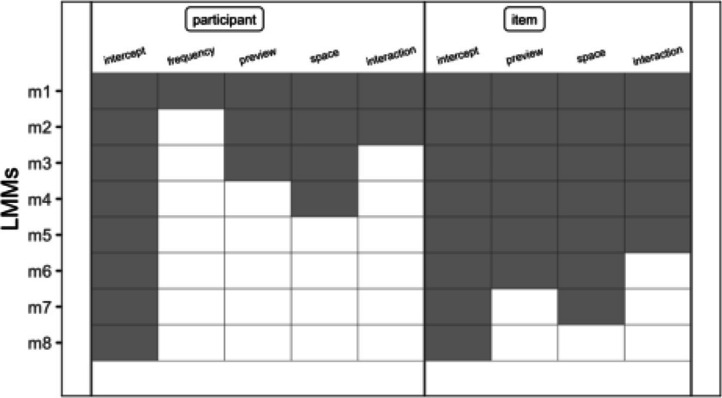
Model trimming for skipping probability

**Fig. 11:**
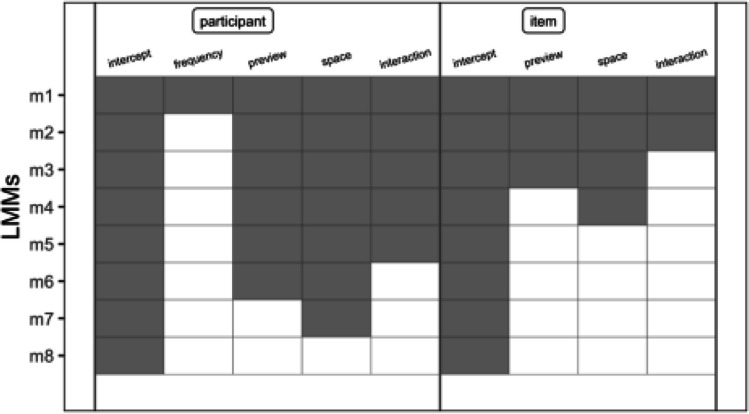
Model trimming for refixation probability

**Fig. 12:**
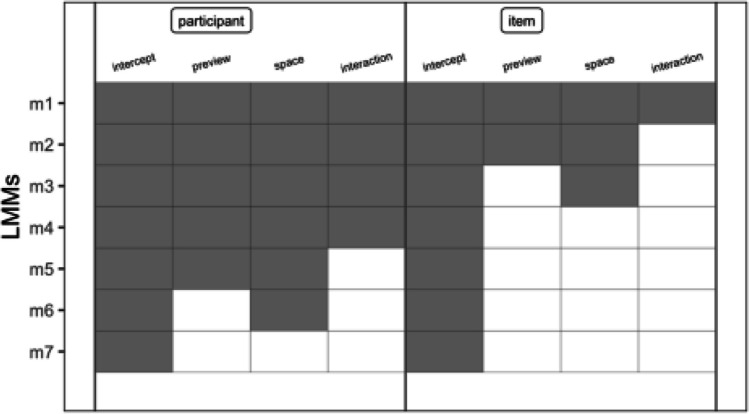
Model trimming for initial landing position

**Fig. 13:**
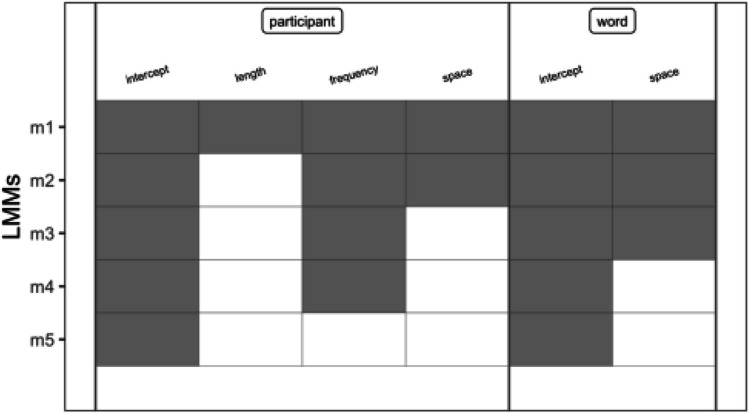
Model trimming for first fixation duration

**Fig. 14:**
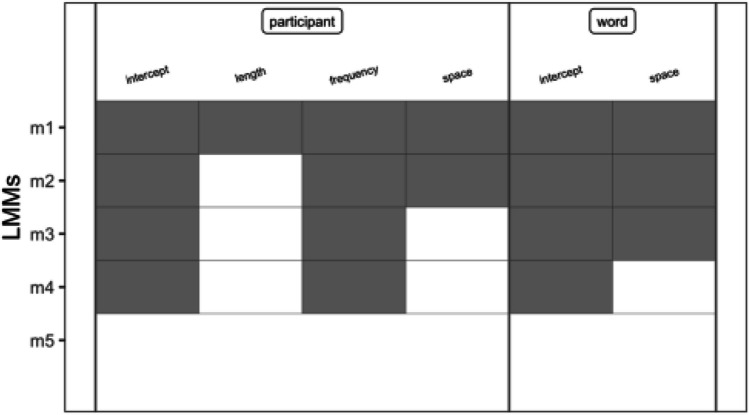
Model trimming for single fixation duration

**Fig. 15:**
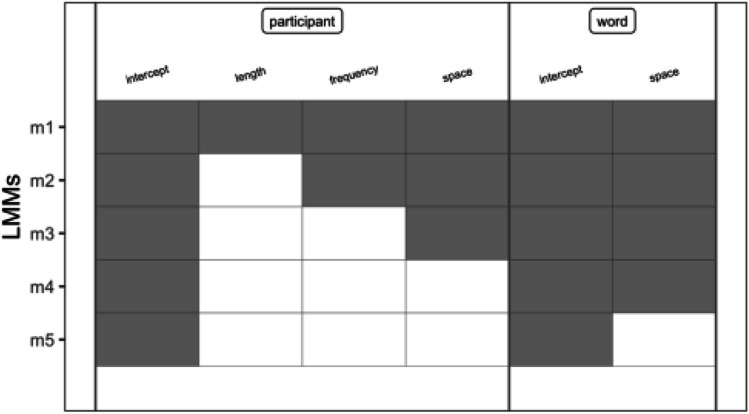
Model trimming for gaze duration

**Fig. 16:**
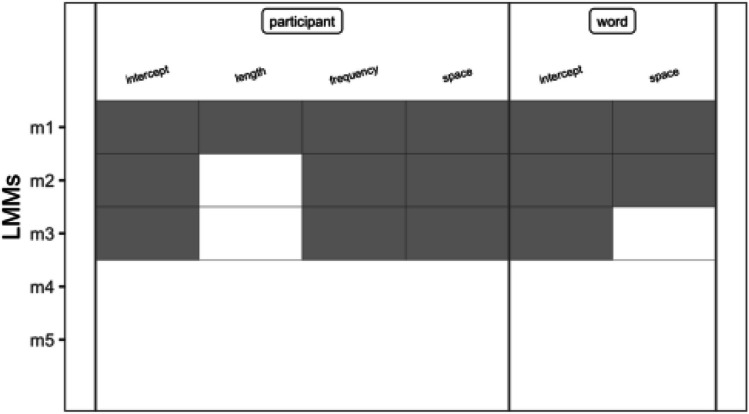
Model trimming for total viewing time

**Fig. 17:**
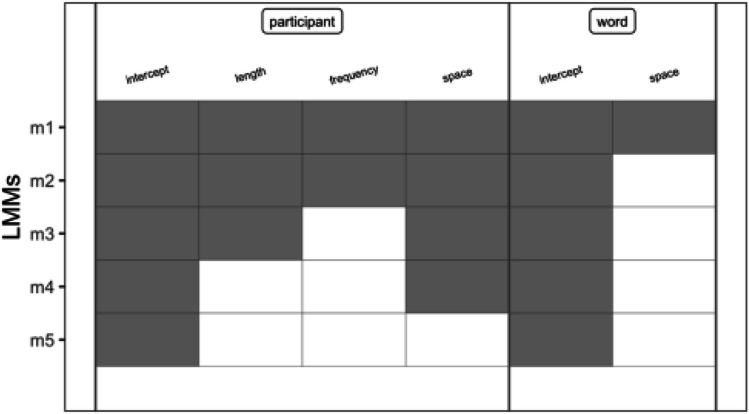
Model trimming for skipping probability

**Fig. 18:**
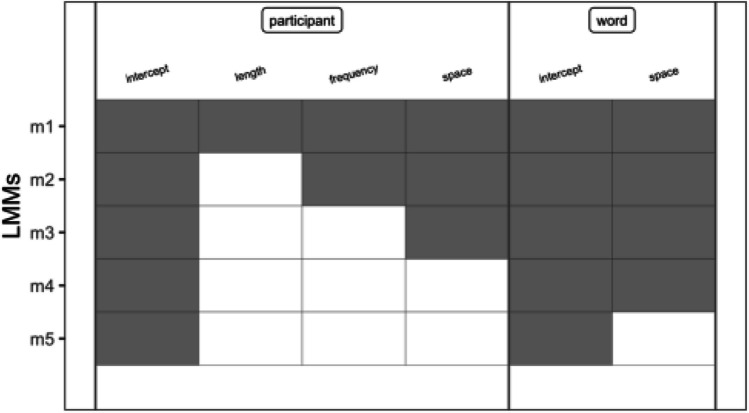
Model trimming for refixation probability

**Fig. 19:**
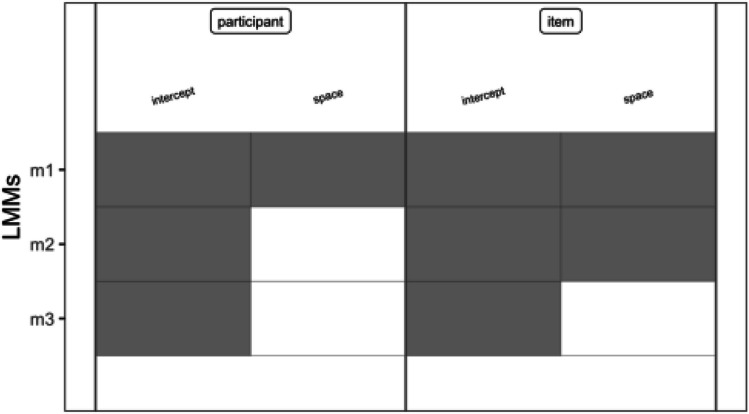
Model trimming for undersweep probability

## Appendix A

**Modeling trimming process for Experiment 1**

This appendix contains tile plots documenting the model trimming procedure used for each dependent measure in Experiment 1. The first model was the model with maximum random effect structure. Random effect structure was iteratively trimmed until model convergence was achieved.

## Appendix B

**Sentence-level analysis for Experiment 1**

**Analysis**

This appendix includes the sentence-level analysis for Experiment 1 using data from the valid preview condition alone. For the sentence-level analysis, we calculated *Number of Fixations *(total number of fixations made whilst reading a sentence), *Average Fixation Duration *(average duration of all fixations made whilst reading a sentence), Average Saccade Amplitude (average amplitude of all saccades made whilst reading a sentence), and *Total Time *(total time spent reading a sentence). We constructed linear mixed-effect models (LMMs) for all dependent measures to provide inferential statistics. LMMs were constructed using the *lme4* (Version 1.1-29; Bates et al., 2014) and *lmerTest* (Version 3.1-3; Kuznetsova et al., 2017) packages in R version 4.1.2 (R core team, 2021). Generalized linear mixed-effect models were constructed with Poisson distribution for count measures (i.e., Number of Fixations). Letter spacing was entered using successive differences contrast (Schad et al., 2020). Following Barr et al. (2013)’s suggestion, we started from models with the maximal random effect structure and trimmed the models until convergence was achieved.

## Results

Descriptive statistics for the dependent measures are summarized in Table 5, whereas statistics from (G)LMMs are summarized in Table 6. Letter spacing had significant effects on number of fixations, average fixation duration, and average saccade amplitude. These effects were significant across all three levels of letter spacing and were manifested by readers making more fixations, having shorter fixation durations, and making longer saccades when letter spacing was increased. Moreover, despite variations in number of fixations, average fixation duration, and average saccade amplitude, readers spent similar total time reading sentences across letter spacing conditions.

Results of Experiment 1 provided valuable insights into how crowding, when manipulated by means of letter spacing, affects reader’s eye movements during natural reading. The sentence-level results showed a trade-off between fixation duration and number of fixations: As letter spacing increased, readers made shorter yet more fixations whilst reading the sentences. These results replicated the previously observed letter spacing effects on global reading parameters (Perea et al., 2016; Perea & Gomez, 2012; Slattery & Rayner, 2013) and also demonstrate a reader’s flexibility in adapting to variations in text configuration.

**Table 5:** Descriptive statistics for dependent measures in Experiment 1

Letter spacing	Num. of Fixations	Avg. Fixation Duration	Avg. Saccade Amplitude	Total Time
Condensed	13.1 (0.43)	231.41 (4.87)	2.1 (0.08)	3407.99 (141.27)
Standard	13.54 (0.47)	226.54 (4.76)	2.43 (0.08)	3465.19 (140.34)
Wide	14.01 (0.49)	218.39 (4.26)	2.77 (0.09)	3503.03 (141.59)

Standard errors are provided in parentheses. Temporal measures were measured in milliseconds. Saccade amplitude was measured in visual angle

**Table 6:** (G)LMM results for dependent measures in Experiment 1

Fixed Effect	Statistics	Num. of Fixations	Avg. Fixation Duration	Avg. Saccade Amplitude	Total Time
Intercept	Estimate	2.58	5.4	0.84	8.1
	Std. Error	0.03	0.02	0.03	0.04
	t/z-value	**75.84**	**269.53**	**26.32**	**202.71**
Wide – Standard	Estimate	0.03	-0.03	0.13	0.01
	Std. Error	0.02	0.01	0.01	0.01
	t/z-value	**2.18**	**-5.15**	**10.65**	1
	*p*-value	**.029**	**<.001**	**<.001**	.32
Standard – Condensed	Estimate	0.03	-0.02	0.15	0.02
	Std. Error	0.02	0.01	0.01	0.01
	t/z-value	**2.08**	**-3.34**	**12.03**	1.52
	*p*-value	**.037**	**.001**	**<.001**	.128

All dependent measures were log-transformed. Significant effects are indicated in **bold**

## Appendix C

**Model trimming process for Experiment 2**

This appendix contains tile plots documenting the model trimming procedure used for each dependent measure in Experiment 2. The first model was the model with maximum random effect structure. Random structure was iteratively trimmed until model convergence was achieved.
